# Huanglongbing and Foliar Spray Programs Affect the Chemical Profile of “Valencia” Orange Peel Oil

**DOI:** 10.3389/fpls.2021.611449

**Published:** 2021-04-06

**Authors:** Xiuxiu Sun, Huqing Yang, Wei Zhao, Elise Bourcier, Elizabeth A. Baldwin, Anne Plotto, Mike Irey, Jinhe Bai

**Affiliations:** ^1^USDA/ARS Horticultural Research Laboratory, Fort Pierce, FL, United States; ^2^Zhejiang A & F University, Hangzhou, China; ^3^Southern Gardens Citrus Nursery, Clewiston, FL, United States

**Keywords:** citrus greening disease, nutritional spray, insect vector control, cold pressed oil, volatile organic compounds

## Abstract

Florida orange trees have been affected by huanglongbing (HLB) for more than a decade. To alleviate disease-caused tree decline, maintain fruit productivity, and reduce disease transmission, enhanced foliar spray programs combining vector control and nutritional supplementation have been applied to healthy and diseased trees. The aim of this research was to discover if the various foliar sprays affect fruit peel oil chemical components. In this study, “Valencia” orange trees, with or without HLB (HLB±), were treated with the grower standard program (control, C) or one of four proprietary enhanced foliar spray programs (N1, N2, N3, and N4) over 16 months. Compared with HLB−, HLB+ samples had lower concentrations of typical peel oil components, including valencene, octanal, and decanal, and were abundant in oxidative/dehydrogenated terpenes, such as carvone and limonene oxide. However, limonene, the dominant component, was not affected by any treatment. Control and three out of four enhanced foliar spray programs, N2, N3, and N4, had very little influence on the chemical profiles of both HLB− and HLB+ samples, while N1 treatment greatly altered the chemical profile of HLB+ samples, resulting in peel oil similar to that of HLB− samples.

## Introduction

Huanglongbing (HLB, or citrus greening), associated with the bacterium *Candidatus Liberibacter* asiaticus (*C*Las), is a very destructive citrus disease and is vector-transmitted by the Asian citrus psyllid (ACP; *Diaphorina citri* Kuwayama). Once infected, trees rapidly decline and eventually die (Bové, 2006; Gottwald et al., 2020). Before the severe decline, HLB-infected trees still produce fruits, however, with decreased yield and poor quality. Fruits are typically small, irregularly shaped, with a thick, pale peel that remains green at the stylar end (Baldwin et al., 2014; Chen et al., 2016; McCollum et al., 2016). Fruits and juice from HLB-diseased trees are also associated with low soluble sugar and ethyl butanoate, as well as high acid, limonoid, and flavonoid content, resulting in less sweet and fruity flavor with more sour and bitter taste (Dala-Paula et al., 2018).

Furthermore, HLB causes an increase in pre-harvest fruit drop, which contributes to the reduction in yield (Baldwin et al., 2014; McCollum et al., 2016). This premature fruit drop may be exacerbated by *Lasiodiplodia theobromae* (previously known as *Diplodia natalensis*), the causal organism of citrus stem end rot (SER), which infects citrus fruits under the calyx abscission zone (Zhao et al., 2015, 2016). HLB has spread over all major citrus growing regions of Florida and has been the primary cause of $7.8 billion in lost revenue and more than 7,500 jobs in the Florida citrus industry since 2006 (Hodges and Spreen, 2012). The orange production of Florida was estimated at 45 million boxes for the 2017–2018 season, which represents a decline of more than 80% from the state’s historic peak citrus production.

Previous research showed remission of citrus decline is more likely if trees remain vigorous by reducing stress caused by nutrient deficiencies, with or without application of plant growth regulators, to help host defense against HLB (Spann and Schumann, 2009; Stansly et al., 2014; Li et al., 2016). Several reports indicated that the application of mineral fertilizers, such as Zn, Cu, and Fe, alleviated the symptoms of HLB symptomatic trees and restored their productivity (Nwugo et al., 2013; Zhang et al., 2016). These favorable results were attributed to plant health maintenance and the promotion of root growth. In certain nutrient/systemic acquired resistance (SAR) programs, salicylic acid (SA) and/or its analogs were applied as foliar amendments to help the host defend against HLB by activating the SAR pathway. A combination of psyllid management and foliar nutrition/SAR sprays maintained tree health and productivity despite the presence of HLB (Rouse et al., 2012). However, any positive effects these treatments have on disease expression or fruit yield remain to be demonstrated (Stansly et al., 2014). In fact, no conclusive study has been conducted demonstrating how to control HLB by inducing plant defense (Li et al., 2016).

Citrus peel oil is the first byproduct obtained during the processing of citrus fruits and is widely used in foods, perfumery, and cosmetics (Dharmawan et al., 2009; Gonzalez-Mas et al., 2019). Citrus oil is also used as a cleaner, solvent, fungicide, and even aromatherapy material for humans (Dugo and Mondello, 2011). Citrus oil possesses strong insecticidal and biochemical activities (Oyedeji et al., 2020), as well as antimicrobial and antioxidant activities (Fancello et al., 2020; Oikeh et al., 2020). The essential oil from Mandarin revealed the inhibitory effects against *Staphylococcus aureus* (Song et al., 2020). A commercial citrus essential oil showed antimicrobial activity against *Escherichia coli* (Ambrosio et al., 2020). In addition, the Blanco peel essential oil exhibited potential for the treatment of skin acne (Hou et al., 2019). The volatile composition of citrus peel oil has also been extensively studied (Minh Tu et al., 2002; Njoroge et al., 2003; Dugo and Mondello, 2011; Liu et al., 2012; Lin et al., 2019). Aldehyde composition is the most important factor to evaluate citrus peel oil quality, and a better-quality oil usually has a higher concentration of aldehydes (Xu et al., 2017b). Aldehydes are known for their distinctly potent fragrances and are often main contributors to the overall flavor of an essential oil, and limonene is the major volatile compound in citrus peel oils (Spadaro et al., 2012). The quality of citrus oil is frequently affected by the climatic condition, disease, and harvest maturity (Vekiari et al., 2002; Zouaghi et al., 2019). However, there has been little research concerning the impact of HLB and nutritional/insecticide sprays on citrus peel oil volatiles. The objective of this study was to investigate the effect of enhanced foliar spray programs on volatile components of cold pressed oil from HLB+ and HLB− Valencia orange peel.

## Materials and Methods

### Chemicals

Standards were obtained from the following sources: octanal, nonanal, decanal, terpinyl aldehyde, (*E*,*E*)-2,4-decadienal, geranial, neral, octanol, linalool, citronellol, nerol, α-pinene, myrcene, and β-phellandrene were purchased from Aldrich (Milwaukee, WI, United States); valencene was bought from Bedoukian (Danbury, CT, United States); undecanal from Analabs (North Haven, CT, United States); sabinene was obtained from Treatt (Lakeland, FL, United States); δ-3-carene was from K&K (Royse City, TX, United States); terpinen-4-ol from Advanced Biotech (Totowa, NJ, United States); and α-humulene from Fluka (Buchs, Switzerland).

### Field Management and Foliar Spray Programs

Experiments were carried out on a commercial block of *Citrus sinensis* (L.) Osbeck cv. Valencia orange on “Swingle” citrumelo (*Citrus paradisi* × *Poncirus trifoliata*) rootstock planted in 2,000 at 3.7 m between trees and 7.3 m between rows at the Southern Garden Citrus Nursery grove in Clewiston, FL (26°45′N; 80°56′W). The training system was round shaped, and average tree height was about 4.75 m. At least one border tree between two different foliar spay programs was provided. The block consisted of 99 trees, and about half of the trees were HLB affected based on a visual diagnose in 2010 by the leaf, fruits and canopy symptoms (Bové, 2006). The trees had received common cultural practices and the grower’s standard pest and disease management before the initiation of the trial in January 2012.

HLB+ and HLB− trees, 30 each, uniform in canopy size, were selected for the study by using a split-plot design with three replicates. Each plot consisted of two subplots, HLB+ and HLB−, and each subplot consisted of two trees. Each plot was treated with one of the following five foliar spray programs for over 16 months from the beginning of 2012 to May 2013, each program included multiple applications, and each application contained macro- (N, P, and K) and micro- (Ca, Mg, Zn, B, Fe, and Mo) nutrients and vector control agents (chlorpyrifos, imidacloprid, abamectin, fenpropathrin, malathion, ζ-cypermethrin, and mineral oil), independently or in combination. The control (C) was the grower standard (Table 1) with eight sprays from April 4, 2012 to May 14, 2013. Each foliar spray program (N1, N2, N3, and N4) was featured by recommendation of one or more nutrient suppliers or grower groups. The exact composition of each foliar spray is proprietary. The common enhancement was an increase in spray times from 8× in the control to 10× in N1 and N4, 11× in N3, and 12× in N2. All N1–N4 had a late dormant/spring flush in Feb 2012, in comparison with the earliest spray for control, which was April 4, 2012 (Table 1).

**TABLE 1:** Chemicals/agents used for the grower standard/control (C) foliar spray program and the application regime.

Spray product	Active ingredient	Function	Company
**A. Characteristics of chemicals/agents used for foliar application**			
Nutri-Phite Magnum 2-40-16	N, P, K	Macronutrients	Verdesian Life Sciences, Cary, NC, United States
Dual Phos 14-7-7	N, P, K	Macronutrients	Griffin Fertilizer Company, Frostproof, FL, United States
Potassium nitrate 3-0-11	N, K	Macronutrients	Haifa North America, Altamonte Springs, FL, United States
Citrite 779	N, Mg, Zn, Fe	Macro/Micronutrients	Chemical Dynamics, Inc., Plant City, FL, United States
Soar Bloom	Ca, Mg, B, Mo	Micronutrients	Chemical Dynamics, Inc., Plant City, FL, United States
Solubor	B	Micronutrient	U.S. Borax, Boron, CA, United States
Nitro-Gold Nut # 1110243	Fe, Mn, Zn	Micronutrient	Southern Ag, Palmetto FL, United States
Whirlwind	Chlorpyrifos	Insecticide	Helena Chemical Co., Collierville, TN, United States
Drexel 80/20 surfactant	Alcohol ethoxylates/glycols	Adjuvant	Drexel Chemical, Memphis, TN, United States
Humac/Surfactant	Alcohol ethoxylates/glycols	Adjuvant	Drexel Chemical, Memphis, TN, United States
435 Spray Oil	Mineral oil	Insecticide	Drexel Chemical, Memphis, TN, United States
Imidacloprid 2F	Imidacloprid	Insecticide	Control Solutions, Inc., Pasadena, TX, United States
Agri-Mek SC	Abamectin	Insecticide	Syngenta, LLC, Vero Beach, FL, United States
Danitol	Fenpropathrin	Insecticide	Valent United States Corporation, Longwood, FL, United States
Malathion	Malathion	Insecticide	Griffin Fertilizer Company, Frostproof, FL, United States
Portal Miticide/Insecticide	Mitochondrial Electron Transport Inhibitor	Insecticide	Nichino America, Inc., Wilmington, DE, United States
Mustang	Zeta-cypermethrin	Insecticide	FMC Corporation, Philadelphia, PA, United States
Nu Cop	Cu	Fungicide	Albaugh Inc., Ankeny, IA, United States
Gem 500 SC	Trifloxystrobin	Fungicide	Bayer, Pittsburgh, PA, United States
Kocide 3000	Cu	Fungicide	Kocide LLC, Houston, TX, United States
**B. Foliar application regime**			
**Application time (phenologic phase)**	**Spray product**	**Dose (amount per ha)**	
4-Apr-2012	Nutri-Phite	2.34	L
(bloom spray)	Soar Bloom	4.68	L
	Kocide 3000	2.24	kg
	Humac/Surfactant	0.56	kg
19-Jun-2012	435 Spray oil	28.06	L
(1st summer oil spray)	Kocide 3000	2.24	kg
	Liquid Potassium Nitrate	42.09	L
	Nutri-Phite Magnum 2-40–16	2.34	L
	Nitro-Gold Nut #1110243	7.02	L
	Imidacloprid 2F	0.56	kg
	Agri-Mek SC	0.15	kg
2-Aug-2012	Gem 500 SC	0.18	kg
(2nd summer oil spray)	Kocide 3000	2.24	kg
	Liquid potassium nitrate	42.09	L
	Citrite 779	7.02	L
	Whirlwind	5.85	L
	Drexel 80/20 Surfactant	0.56	kg
20-Sep-2012	435 Spray oil	28.06	L
(3rd summer oil spray)	Kocide 3000	2.24	kg
	Liquid potassium nitrate	42.09	L
	Nutri-Phite Magnum 2-40-16	2.34	L
	Nitro-Gold Nut #1110243	7.02	L
	Imidacloprid 2F	1.12	kg
	Agri-Mek SC	0.30	kg
17-Dec-2012	Danitol	1.12	kg
(dormant spray)	Drexel 80/20 surfactant	0.21	kg
25-Jan-2013	Dual Phos 14-7-7	65.48	L
(flash spray)	Malathion	1.75	L
	80/20	0.56	kg
5-Mar-2013	Soar Bloom	4.68	L
(prebloom spray)	Nutri-Phite	2.34	L
	Potassium Nitrate 3-0-11	42.09	L
	Nu Cop	2.24	kg
	Portal	4.68	L
	80/20	0.56	kg
14-May-2013	Kocide 3000	2.24	kg
(postbloom spray)	Potassium Nitrate 3-0-11	42.09	L
	Nutri-Phite	2.34	L
	Nitro-Gold Nut #1110243	7.02	L
	Mustang	0.30	kg
	80/20	0.56	kg
	Solubor	1.12	kg

### Fruit Harvest and Sample Processing

Fruits were harvested on April 28, 2014. For each replicate, approximately 38.5 kg (56 fruits) were harvested from the two trees. Fruits were washed and processed by using a standard processing method with a JBT extractor (JBT^§^ FoodTech, Lakeland, FL, United States) at premium setting (Bai et al., 2013). Juice samples were collected for *C*Las titer testing; and “frit” peel tissues, which are located in around of the stem end and are rich in peel oil, were also collected for peel oil extraction. Briefly, the frit peel tissues were cold pressed with a manual oil extractor, the collected emulsion allowed to settle for 30 min, and then the top oil layer was collected and centrifuged at 3,500 × *g* at 25°C for 15 min. Finally, the peel oil supernatant was collected and stored at −20°C for analysis. To protect the oil samples from oxidation, the headspace of the sample vial was flushed with nitrogen gas before air-tight capping.

### DNA Extraction and qPCR Detection of *Candidatus Liberibacter* asiaticus in Leaves and Fruit Juice

For leaf samples, 10 leaves from each tree were randomly taken on the same day as fruit harvest, and DNA was extracted from 100 mg of the midrib tissues by following Li et al.’s (2006) procedures. Primers targeting *C*Las 16S rDNA (Li primers) were used (Li et al., 2006; Baldwin et al., 2018); and TaqMan qPCR was performed in a 7500 Real-Time PCR system (Applied Biosystems, Inc., Carlsbad, CA, United States).

For fruit juice samples, DNA was extracted from 500 μl of orange juice sample using a modified cetyl trimethylammonium bromide (CTAB) method (Zhao et al., 2018), and *C*Las level was quantified by qPCR as previously described (Zhao et al., 2018) using primers targeting *C*Las hyv1 (LJ primers) (Morgan et al., 2012); SYBR Green qPCR was performed in a 7,500 Real-Time PCR system (Applied Biosystems). The default melt curve (disassociation) stage was continued after the 40 cycles of PCR. Quantification cycle (Cq) values were analyzed using ABI 7,500 Software version 2.0.6 (Applied Biosystems) with a threshold setting of 0.02 and automated baseline settings.

### Peel Oil Volatile Composition Analysis

The volatile composition analysis of the oil was carried out using a gas chromatography–mass spectrometry (GC-MS, 6890N GC and 5975 MS, Agilent Technologies, Santa Clara, CA, United States) system equipped with a split/splitless injector and a DB-5 capillary column (60-m length, 0.25-mm diameter, and 1-μm film thickness; J&W Scientific, Folsom, CA, United States). The injector and detector temperature was 260°C. The injection volume was 1 μl with a split ratio of 40:1. The oven conditions were 40°C (0.5 min) and then 4°C⋅min^–1^ to 225°C (held for 13.25 min) for a total run time of 60 min. Helium was used as carrier gas at flow rate of 1.5 ml⋅min^–1^. Inlet, ionizing source, and transfer line were kept at 250, 230, and 280°C, respectively. The mass spectrometry data were recorded in the scan mode from 40 to 400 m/z at 2 scans⋅s^–1^ with an ionization energy of 70 eV.

### Identification of Volatile Compounds

Data were collected using the ChemStation G1701 AA data system (Hewlett-Packard, Palo Alto, CA, United States). A mixture of C-5 to C-18 *n*-alkanes was run at the beginning of each day to calculate retention indices (RIs) (Bai et al., 2014). The volatile components were identified by matching their spectra with those from NIST/EPA/NIH Mass Spectral Library (NIST 14) and authentic volatile compound standards, as well as by comparing their RIs with corresponding literature data (Lota et al., 2002; Deng et al., 2017). Quantification of major peel oil volatile components (limonene, hexanol, hexanal, linalool, etc.) was conducted by using a peak size vs. concentration curve built by a series of diluted standard solutions (Bai et al., 2002).

### Statistical Analyses

Data presented were the mean values of three biological replicates. Statistical analysis was performed with JMP (version 11.2.2; SAS Institute, Cary, NC, United States). Differences were tested for significance by using a one-way analysis of variance (ANOVA), incorporating a split-plot design. Mean separations were examined by Tukey’s HSD tests with the significance level at 0.05. For both principal component analysis (PCA) and hierarchical cluster analysis, the complete dataset including all replicates was performed. To visualize quantitative results, a heatmap was designed to present all volatile compounds detected in 10 combinations of foliar spray programs × tree types with the relative amounts in each chemical compound.

## Results

### *Candidatus Liberibacter* asiaticus Infection Severity of Leaves and Juice

The qPCR analysis of leaves confirmed the *C*Las uninfected status of “healthy” trees (HLB−), with high Cq values (>38.29), while diseased trees (HLB+) had low Cq values (<21.76;Table 2). The cutoff Cq value between HLB+ and HLB− for Li primers has been determined to be 32 (Zhao et al., 2018).

**TABLE 2:** Quantification cycle (Cq) values of *C*Las in “Valencia” orange leaf midrib and fruit tissue samples affected by Huanglongbing (HLB) and foliar spray programs.

Foliar spray program ^*z*^	HLB−	HLB+
*C*Las 16S rDNA in leaf midrib		
C	38.62 ± 3.38^a,y^	20.75 ± 0.68^b^
N1	40.00 ± 0.00^a^	21.76 ± 0.66^b^
N2	38.66 ± 1.99^a^	21.24 ± 0.71^b^
N3	39.36 ± 1.57^a^	21.72 ± 0.73^b^
N4 ANOVA significance	38.29 ± 2.00^a^***	21.33 ± 0.20^b^
*C*Las hyv1 in orange juice		
C	33.45 ± 1.82^b,c^	28.97 ± 0.97^c,d^
N1	40.00 ± 0.00^a^	29.63 ± 0.52^c^
N2	32.07 ± 2.99^c^	27.24 ± 1.37^d^
N3	38.41 ± 2.75^a,b^	27.90 ± 0.95^d^
N4 ANOVA significance	29.03 ± 0.97^c^***	27.57 ± 2.52^d^

^*z*^Spray treatments: C, control; N1–N4, four experimental foliar nutrient spray programs. ^*y*^Means ± standard deviations within the same block with the same letter are not significantly different by Tukey’s honestly significant difference (HSD) (*p* < 0.05). ***indicates significant at *p* < 0.001.

Because the *C*Las titer in orange juice is much lower than in leaf tissue, we used another pair of primers targeting at *C*Las hyv1, which has more copies in the genome than that of rDNA (Morgan et al., 2012). With these primers, a cutoff Cq value of 29 was determined to differentiate between HLB+ and HLB− (Zhao et al., 2018). Similar to healthy leaf tissue, healthy juice had Cq values higher than the cutoff value, with N4 HLB− right at the cusp (Cq = 29.03) (Table 1). For HLB+ juice, N1 treatment resulted in a higher Cq value (29.63) than N2 (27.24) and N3 (27.90) treatments and slightly higher than cutoff value, indicating that N1 may have some effects on reducing *C*Las titer in juice (Table 2).

### The Effect of Huanglongbing on the Volatile Profile of “Valencia” Citrus Peel Oil

A total of 53 volatile compounds were identified in peel oil samples (Table 3), including seven monoterpenes, 14 sesquiterpenes, 13 terpene oxides, 10 aldehydes, 8 alcohols, and 1 ketone. Monoterpenes were predominant in all the “Valencia” citrus peel oil samples with limonene accounting for the major constituent (89.74–90.48%, Table 3). In general, there were more differences among all the treatments within HLB+ fruits (23 volatile compounds total) than among all the treatment within HLB− fruits (hexanal only).

**TABLE 3:** Chemical compositions of peel oils of “Valencia” oranges affected by Huanglongbing (HLB) and foliar spray programs N1, N2, N3, and N4, with C as control fertilization program.

Peak	Compound	RI	HLB−					HLB+					ANOVA significance
			C^*z*^	N1	N2	N3	N4	C	N1	N2	N3	N4	
1	Hexanal	795	0.02^*a,by*^	0.01^b,c^	0.01^a,b,c^	0.01^b,c^	0.02^a^	0.01^c^	0.01^c^	0.01^c^	0.01^c^	0.01^c^	***
2	Hexanol	868	0.01^b^	0.01^a,b^	0.01^a,b^	0.01^a^	0.01^a,b^	0.01^a^	0.01^a,b^	0.01^a,b^	0.01^a,b^	0.01^a,b^	*
3	α-Pinene	954	1.11	1.14	1.10	1.11	1.16	1.15	1.11	1.08	1.07	1.11	NS
4	Sabinene	993	0.66^a,b^	0.48^b^	0.63^a,b^	0.74^a,b^	0.73^a,b^	0.99^a^	0.61^a,b^	0.88^a,b^	0.73^a,b^	0.87^a,b^	*
5	Myrcene	1,002	2.56	2.59	2.54	2.55	2.68	2.59	2.49	2.45	2.41	2.51	NS
6	Octanal	1,012	0.72	0.60	0.69	0.62	0.70	0.58	0.67	0.47	0.56	0.54	NS
7	α-Phellandrene	1,025	0.11	0.11	0.10	0.10	0.11	0.10	0.10	0.10	0.09	0.09	NS
8	δ-3-Carene	1,032	0.14	0.13	0.11	0.10	0.15	0.13	0.08	0.14	0.11	0.13	NS
9	Limonene	1,050	90.07	90.02	89.96	89.99	89.74	89.74	89.95	90.19	90.48	90.23	NS
10	β-Phellandrene	1,063	0.21	0.25	0.22	0.20	0.21	0.21	0.17	0.23	0.18	0.20	NS
11	Octanol	1,076	0.15	0.17	0.17	0.18	0.15	0.19	0.17	0.16	0.19	0.15	NS
12	Linalool	1,109	0.83	0.85	0.83	0.84	0.88	0.92	0.90	0.84	0.88	0.87	NS
13	Non-anal	1,112	0.15	0.14	0.15	0.14	0.15	0.14	0.16	0.13	0.13	0.13	NS
14	(*E*)-*p*-Mentha-2,8-dien-1-ol	1,139	0.01^c^	0.01^c^	0.01^b,c^	0.01^b,c^	0.01^b,c^	0.01^b,c^	0.01^b,c^	0.02^a^	0.01^a,b^	0.01^a,b^	***
15	(*Z*)-*p*-Mentha-2,8-dien-1-ol	1,153	0.01^a,b^	0.01^a,b^	0.01^a,b^	0.01^a,b^	0.01^a,b^	0.00^c^	0.01^a,b^	0.01^a^	0.01^a,b^	0.00^b,c^	***
16	(*E*)-Limonene oxide	1,157	0.02^a^	0.02^a^	0.02^a^	0.02^a^	0.02^a^	0.02^a^	0.02^a^	0.02^a^	0.02^a^	0.02^a^	*
17	Citronellal	1,161	0.13	0.12	0.12	0.12	0.13	0.14	0.11	0.12	0.13	0.14	NS
18	Non-anol	1,173	0.01	0.01	0.01	0.01	0.01	0.01	0.01	0.01	0.01	0.01	NS
19	Camphor	1,175	0.01	0.01	0.01	0.01	0.01	0.01	0.01	0.01	0.01	0.01	NS
20	Terpinen-4-ol	1,199	0.02^c^	0.02^c^	0.02^b,c^	0.02^b,c^	0.02^b,c^	0.02^a,b,c^	0.02^a,b,c^	0.03^a^	0.02^a,b,c^	0.03^a,b^	**
21	Decanal	1,210	0.95	0.93	0.97	0.92	0.94	0.85	1.00	0.82	0.82	0.80	NS
22	Nerol	1,230	0.04	0.04	0.04	0.04	0.04	0.05	0.04	0.05	0.05	0.05	NS
23	*p*-Menth-1-en-9-al	1,235	0.05^a,b^	0.05^a,b^	0.05^a,b^	0.05^a,b^	0.04^b^	0.06^a,b^	0.05^a,b^	0.07^a^	0.06^a,b^	0.06^a,b^	*
24	(*Z*)-Carvone	1,237	0.01^a,b,c^	0.01^a,b,c^	0.01^b,c^	0.01^b,c^	0.01^b,c^	0.01^a,b,c^	0.01^c^	0.01^a,b^	0.01^a,b,c^	0.01^a^	**
25	Neral	1,249	0.16	0.15	0.16	0.15	0.16	0.16	0.16	0.14	0.14	0.15	NS
26	Geraniol	1,256	0.03	0.04	0.04	0.04	0.03	0.04	0.04	0.04	0.04	0.03	NS
27	Carvone	1,261	0.01^b^	0.01^b^	0.01^b^	0.01^b^	0.01^b^	0.02^a,b^	0.01^b^	0.03^a^	0.02^a,b^	0.02^a,b^	**
28	(*E*,*E*)-Decenal	1,265	0.01	0.01	0.01	0.01	0.01	0.01	0.01	0.01	0.01	0.01	NS
29	Geranial	1,275	0.23	0.21	0.23	0.22	0.22	0.23	0.22	0.20	0.20	0.21	NS
30	Perilla aldehyde	1,295	0.05	0.04	0.05	0.05	0.06	0.05	0.04	0.05	0.05	0.05	NS
31	(*E*,*E*)-2,4-Decadienal	1,321	0.02^a,b,c^	0.02^a,b,c^	0.02^a^	0.02^a,b^	0.02^a,b,c^	0.01^c^	0.02^a^	0.01^b,c^	0.01^a,b,c^	0.01^a,b,c^	**
32	α-Terpinyl acetate	1,358	0.04	0.05	0.03	0.04	0.04	0.02	0.03	0.04	0.03	0.02	NS
33	α-Copaene	1,401	0.09^a,b^	0.12^a^	0.10^a,b^	0.09^a,b^	0.09^a,b^	0.07^b^	0.11^a,b^	0.08^b^	0.09^a,b^	0.07^b^	**
34	Dodecanal	1,405	0.23	0.21	0.23	0.21	0.21	0.21	0.24	0.19	0.19	0.19	NS
35	β-Cubebene	1,412	0.14B	0.13	0.15	0.17	0.15	0.19A	0.16	0.21	0.17	0.20	NS
36	(*Z*)-Carveol	1,416	0.04	0.05	0.04	0.05	0.05	0.03	0.04	0.04	0.03	0.03	NS
37	(*E*)-Caryophyllene	1,453	0.06^b^	0.08^a,b^	0.10^a,b^	0.08^a,b^	0.07^b^	0.09^a,b^	0.12^a^	0.11^a^	0.09^a,b^	0.09^a,b^	**
38	(*E*)-β-Farnesene	1,455	0.02^a,b,c^	0.01^c^	0.02^a,b,c^	0.03^a,b,c^	0.03^a,b,c^	0.03^a,b^	0.02^b,c^	0.04^a^	0.02^a,b,c^	0.04^a^	**
39	β-Copaene	1,459	0.16	0.16	0.16	0.17	0.16	0.15	0.17	0.16	0.16	0.15	NS
40	α-Humulene	1,488	0.02	0.02	0.02	0.02	0.02	0.02	0.02	0.03	0.02	0.02	NS
41	γ-Cadinene	1,501	0.02	0.02	0.02	0.02	0.02	0.01	0.02	0.01	0.02	0.01	NS
42	α-Farnesene	1,506	0.06B	0.06	0.06	0.07	0.07	0.08A	0.06	0.07	0.07	0.08	NS
43	Germacrene D	1,513	0.06	0.06	0.04	0.06	0.05	0.05	0.06	0.05	0.05	0.05	NS
44	γ-Selinene	1,515	0.00	0.01	0.03	0.01	0.01	0.01	0.02	0.01	0.01	0.01	NS
45	Valencene	1,524	0.17^b,c^	0.30^a^	0.26^a,b,c^	0.21^a,b,c^	0.18^a,b,c^	0.14^c^	0.29^a,b^	0.22^a,b,c^	0.18^a,b,c^	0.15^c^	**
46	α-Selinene	1,529	0.02^a,b,c^	0.02^a^	0.02^a,b^	0.02^a,b,c^	0.02^a,b,c^	0.01^c^	0.02^a,b^	0.02^a,b,c^	0.02^b,c^	0.01^c^	***
47	δ-Cadinene	1,545	0.11^a,b^	0.13^a^	0.12^a,b^	0.11^a,b^	0.11^a,b^	0.09^b^	0.13^a^	0.10^a,b^	0.10^a,b^	0.09^b^	**
48	Germacrene D-4-ol	1,612	0.02^a^	0.02^a,b^	0.01^a,b^	0.01^a,b^	0.02^a,b^	0.01^a,b^	0.01^a,b^	0.01^a,b^	0.01^a,b^	0.01^b^	*
49	Caryophyllene oxide	1,629	0.02^a^	0.03^a,b^	0.02^a,b^	0.02^a,b^	0.02^a,b^	0.02^a,b^	0.03^a^	0.02^a,b^	0.02^a,b^	0.02^a,b^	*
50	β-Gurjunene	1,663	0.01^a,b^	0.01^a,b^	0.01^b^	0.02^a^	0.01^a,b^	0.01^a,b^	0.01^a,b^	0.01^a,b^	0.01^a^	0.01^a,b^	*
51	α-Sinensal	1,773	0.10	0.10	0.10	0.11	0.11	0.11	0.09	0.10	0.10	0.10	NS
52	Octadecanal	1,828	0.03	0.10	0.04	0.05	0.03	0.05	0.04	0.04	0.04	0.04	NS
53	Nootkatone	1,873	0.04	0.05	0.04	0.04	0.04	0.03	0.04	0.04	0.04	0.03	NS

^*z*^Spray treatments: C, control; N1–N4, four experimental foliar nutrient spray programs. ^*y*^Means within the same row with the same letter are not significantly different by Tukey’s honestly significant difference (HSD) (*p* < 0.05). *, **, and *** indicate significance at *p* < 0.05, 0.01, and 0.001, respectively; no significant effect is denoted by NS.

**FIGURE 1:**
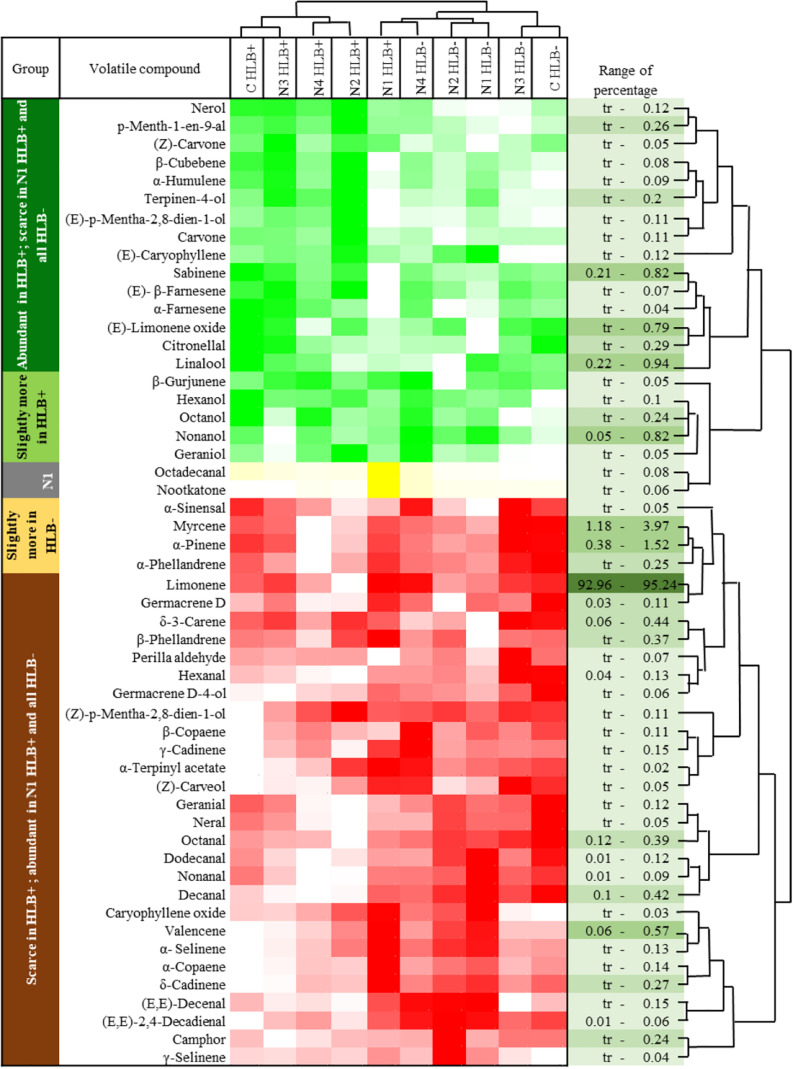
Chemical groups enhanced and suppressed by huanglongbing (HLB) determined by average concentration of volatiles in peel oils extracted from “Valencia” oranges affected by HLB and foliar spray programs (C, control; N1–N4, four different foliar nutrient spray programs). Green and red represent enhanced and suppressed volatiles, respectively. Yellow represents volatiles only enhanced by N1 HLB+ combination. Color density indicates the relative content.

**FIGURE 2:**
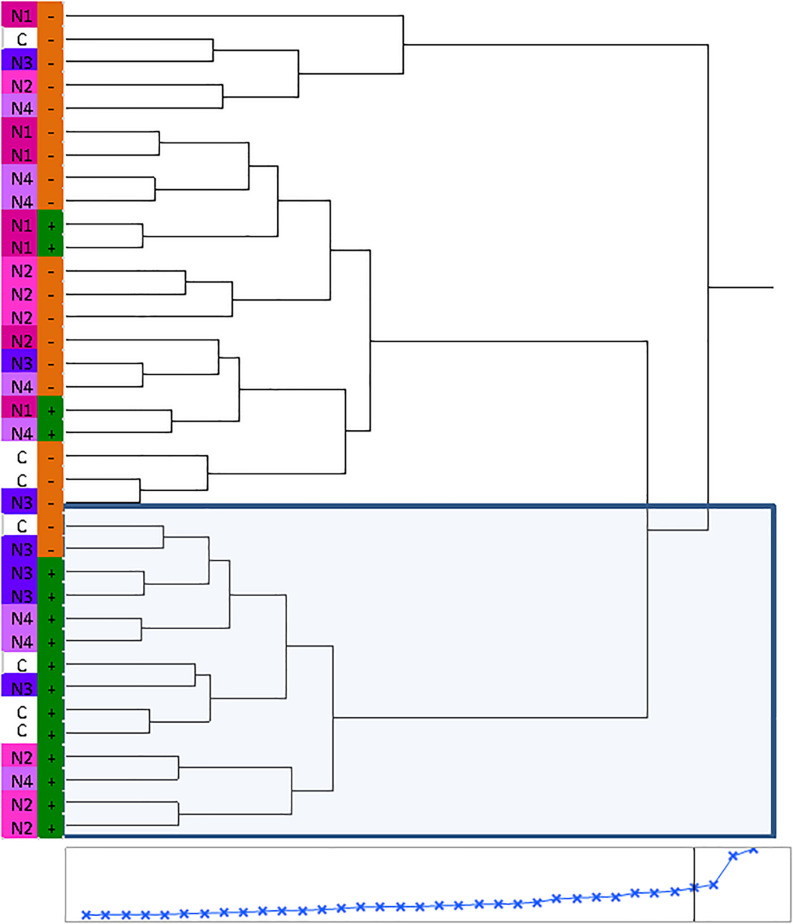
Hierarchical clustering of volatile chemicals in peel oils extracted from “Valencia” oranges affected by Huanglongbing (HLB) and foliar spray programs (C, control; N1–N4, four different foliar nutrient applications; + in green, HLB+; and – in orange, HLB–).

**FIGURE 3:**
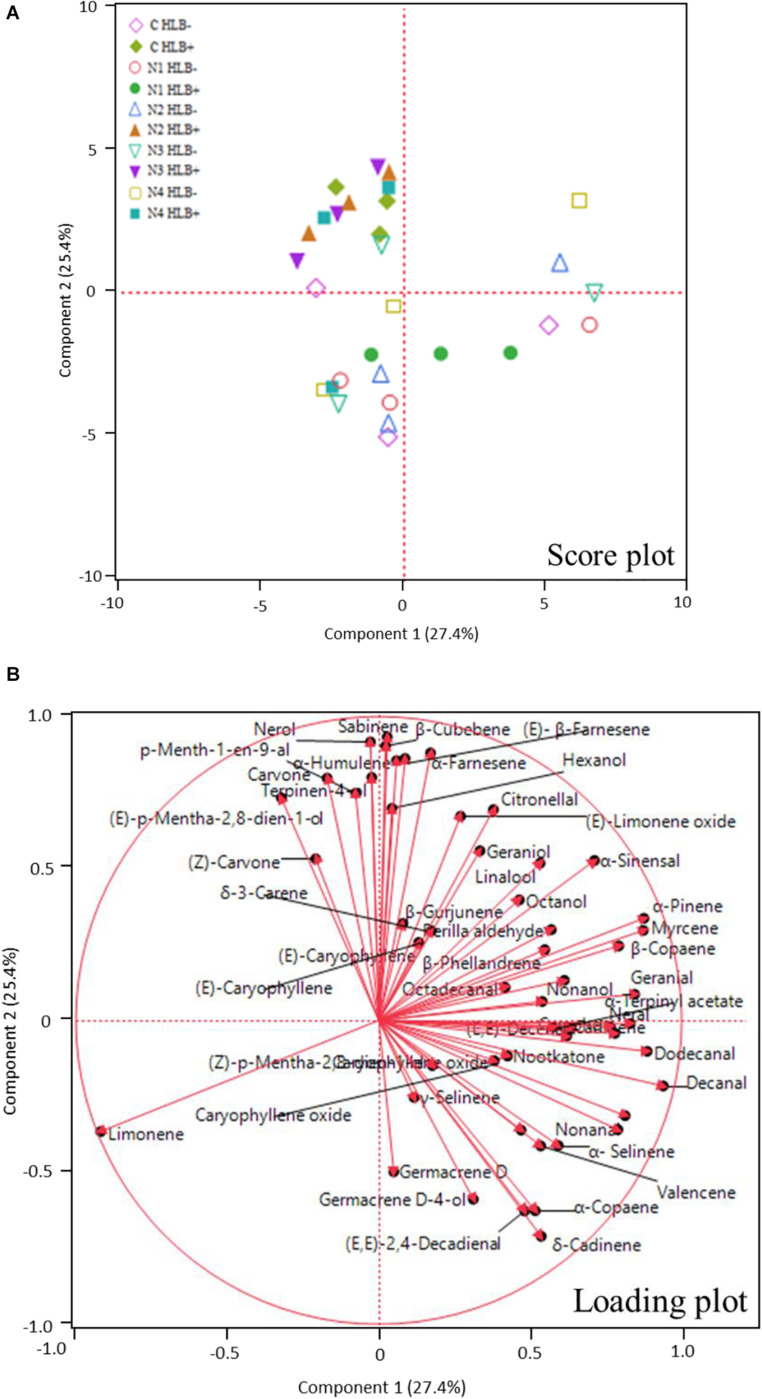
Principle component analysis (PCA) score plot **(A)** and loading plot **(B)** of volatile compounds in peel oils of “Valencia” oranges affected by Huanglongbing (HLB) and foliar spray programs (C, control; N1–N4, four different foliar nutritional sprays).

Huanglongbing disease dramatically affected the volatile profile of citrus fruits (Figures 1, 2). Cluster analysis (Figure 1) showed which compounds were more abundant in HLB− (in red) versus HLB+ (in green) and how the various treatments affected the profile. Hierarchical clustering shows that all the HLB+ N1 samples (dark pink/green) are clustered with the HLB− samples (red) (Figure 2) along with one replicate of HLB+ N4 sample (light purple/green). Analysis by PCA discriminated HLB+ from HLB− samples in both PC1 and PC2 (Figure 3B), explaining 52.8% of the variation in the first two components (Figure 3A). Most of peel oil volatiles from HLB+ samples were on the negative side of PC1 and positive side of PC2 (Figures 3A,B).

### The Effect of Nutritional Spray Treatments on the Volatile Profile of “Valencia” Citrus Peel Oil

The treatment N1 showed the strongest impact on the volatile profile, especially for the HLB+ samples (Table 3 and Figures 1, 2). Treatment N1, for example, reduced (less green) compounds prevalent in HLB+ (e.g., sabinene) and enhanced (more red) some compounds prevalent in HLB− (e.g., α-terpinyl acetate), thus resulting in a profile more closely resembling peel oil from HLB− than oil from HLB+ trees subjected to other treatments. The N1 treatment, which had the highest fruit juice Cq values in both HLB+ and HLB− fruit tissue (Table 2), significantly reduced the concentration of hexanol, sabinene, δ-3-carene, (*E*)-limonene oxide, citronellal, *p*-menth-1-en-9-al, (*Z*)-carvone, and (*E*)-β-farnesene but significantly increased the concentration of (*Z*)-*p*-mentha-2,8-dien-1-ol, decanal, (*E*,*E*)-2,4-decadienal, α-terpinyl acetate, α-copaene, (*E*)-caryophyllene, valencene, α-selinene, δ-cadinene, and caryophyllene oxide (Table 3 and Figure 3B) for HLB+ samples compared with the HLB+ control. Comparing HLB+ with HLB− within N1 treatment showed widespread similarity, with the noticeable exceptions of octadecanal, δ-3-carene, and β-phellandrene, which were more abundant in HLB− than in HLB+ peel oil. All four replicate samples of HLB+ N1 and one replicate sample of HLB+ N4 were positioned on the negative side of PC2, together with most of HLB− samples (Figure 3A).

## Discussion

### Infection Severity of Fruit

A strict ACP control program had been thoroughly enforced since HLB was detected in the citrus orchard throughout the entire foliar spray research period. The qPCR results indicate all HLB− trees remained *C*Las-negative for the duration of the experiments (Table 2). All HLB+ trees remained *C*Las-positive (Table 2), indicating that the enhanced nutritional and ACP vector control applications (such as in Table 1) did not kill *C*Las. However, the qPCR tests using juice samples provided more complex results: in the HLB− trees with N4 foliar spray programs, the Cq value was 29.03 in the juice samples (close to the 29 cutoff value for juice samples determining *C*Las infection by LJ primers), while it was 38.29 in leaf midribs (well above the cutoff value of 32 for leaf *C*Las infection by Li primers) (Table 2). On the other hand, in the HLB+ N1 juice samples, a relatively high Ct value of 29.63 (just above the cutoff value for juice *C*Las infection by LJ primers) was determined, indicating the infection was less severe than in the other HLB+ samples (Table 2). The possible interpretations are as follows: although *C*Las is a phloem-restricted bacterium, and leaf midribs are rich in phloem vessels and thus high in *C*Las titers (Li et al., 2006; Bai et al., 2013), due to the uneven distribution of the pathogen organisms in the tree, leaf samples taken from a tree may test negative for *C*Las, even if some branches are already infected, especially at the early infection stages when only few branches are infected (Bai et al., 2013; McCollum et al., 2016). Although *C*Las titers in fruit juice are much lower than in leaves (Bai et al., 2013; Zhao et al., 2018), juice samples were from fruit harvested from the entire tree, including the infected branches, and thus represent the entire tree status better than a leaf sample. The difficulty of detecting *C*Las in juice samples was overcome by using a pair of primers with high copy numbers in the genome. It has been reported that qPCR using LJ primers targeting *C*Las hyv1 DNA improved the *C*Las detection accuracy in comparison with Li primers (Morgan et al., 2012), because there are more copies of hyv1 gene in the *C*Las genome than 16s rDNA, and therefore, the relative detectable threshold by LJ primers can be reduced by 7–11 cycles for leaf samples (Morgan et al., 2012) and 2–5 cycles for fruit juice samples (Zhao et al., 2018).

### The Effect of Huanglongbing on the Volatile Profile of “Valencia” Citrus Peel Oil

The volatile profiles for both HLB− and HLB+ in this study are similar to what has been reported in previous research (Lota et al., 2002; Njoroge et al., 2003; Huang et al., 2017). Previous research showed that HLB resulted in a significant reduction in aldehydes, peel oil aroma volatiles formed during the normal ripening of HLB− fruits (Kiefl et al., 2018). In this research, the content of aldehydes hexanal, octanal, nonanal, decanal, and dodecanal was also significantly reduced in the HLB+ samples (Table 3 and Figure 1), confirming that HLB negatively affects peel oil quality. Furthermore, similar to previous research, citrus peel oil derived from HLB+ fruits had lower concentrations of citronellal and geranial than oil derived from HLB− samples (Table 3; Xu et al., 2017a). Additionally, some esters, such as α-terpinyl acetate, decreased in HLB+ compared with HLB− samples (Plotto et al., 2008; Baldwin et al., 2010). Conceivably, severe HLB infection could substantially inhibit host secondary metabolism and volatile formation (Xu et al., 2017a).

The oxidative/dihydrogen compounds typical of terpenes, such as carvone and limonene oxide, were significantly higher in HLB+ samples. It is possible that these compounds increased because of the stress induced by HLB. These compounds may have all increased due to oxidative stress, which occurred in trees with severe HLB symptoms, creating greater concentrations of these volatiles (Xu et al., 2017a).

### The Effect of Nutritional/Insecticidal Spray Treatments on the Volatile Profile of “Valencia” Citrus Peel Oil

Florida growers have been using foliar nutritional spray products that often contain micro- and macro-nutrients to compensate for lack of nutrient assimilation due to the HLB disease and compounds that are believed to activate SAR pathways in plants to increase tree defense response (Masaoka et al., 2011; Gottwald et al., 2012). The results in this experiment showed that one of the experimental foliar spray programs, N1, substantially altered the chemical profiles in HLB+ peel oil, and interestingly, the chemical profile turned out to be similar to that of HLB− peel oil. In N1 sprayed HLB+ peel oil, the most significant changes were recovery of valencene and other sesquiterpenes, which were suppressed by HLB (Table 3 and Figure 3). Phylogenetic analysis of plant terpene synthase genes localized *Cstps1* to the group of angiosperm sesquiterpene synthases (Sharon-Asa et al., 2003). Within this group, *Cstps1* belongs to a subgroup of citrus sesquiterpene synthases (Sharon-Asa et al., 2003; Yu et al., 2019). *Cstps1* was found to be developmentally regulated: transcripts were found to accumulate only during fruit maturation, which corresponds to the timing of valencene accumulation in fruits (Sharon-Asa et al., 2003). HLB causes deficiency of valencene and other sesquiterpenes most likely due to the delay of fruit maturation (Dala-Paula et al., 2018), and the recovery of valencene and other sesquiterpenes indicates that the N1 spray program improved tree defense response, through either nutritional improvement, vector control preventing ACP mediated exacerbation of HLB, or a combination of both (Stansly et al., 2014; Li et al., 2016). Table 2 clearly shows that the N1 spray program significantly reduced the *C*Las titer in orange juice sample. Further research is required to confirm if the presence of HLB results in a downregulation of *Cstps1*, and if a N1-like spray program can enhance the recovery.

As the experimental foliar sprays are of proprietary formulation, we do not know the exact difference between N2, N3, and N4. N1, which used two types of Zn and three types of Mg containing chemicals, was the only treatment that showed an improvement in the chemical profile of the peel oil (Table 3 and Figure 3).

Peel oil quality is determined by the proper chemical combination of volatile compounds. Several low−abundant sesquiterpenes, such as valencene, α−sinensal, and β−sinensal, stand out in citrus as important flavor and aroma compounds (Dugo and Mondello, 2011). The profile of terpenoid volatiles in various citrus species and their importance as aroma compounds have been studied in detail (Njoroge et al., 2003; Xu et al., 2017a; Lin et al., 2019), but much is still lacking in our understanding of the physiological, biochemical, and genetic regulation of their production. Proteomic approaches, such as SWATH-MS, could be used to facilitate functional analysis in plant research (Zhu et al., 2020). Proteogenomics combines proteomics, genomics, and transcriptomics and has considerably improved genome annotation in poorly investigated phylogenetic groups for which homology information is lacking and may be a fruitful approach for elucidating the host–pathogen relationship between citrus and HLB with respect to the biosynthesis of volatile components of peel oil (Blank-Landeshammer et al., 2019; Chen et al., 2020).

## Conclusion

In this research, the influence of HLB disease and foliar spray programs on the chemical composition of “Valencia” orange peel oil was investigated. HLB disease altered the volatile profile of “Valencia” orange peel oil in that many terpene compounds were accumulated at a higher level in the HLB+ peel oil, indicating that disease stress up-regulated the terpenoid pathways. In contrast, some key aldehydes in peel oil were suppressed in the HLB+ samples, which may negatively impact peel oil quality. Of the four proprietary foliar spray programs tested in this research, only N1 shifted the chemical profile of HLB+ peel oil to resemble that of HLB− samples.

## Data Availability Statement

The original contributions presented in the study are included in the article, further inquiries can be directed to the corresponding author/s.

## Author Contributions

EAB, MI, AP, and JB: conceptualization. JB, MI, WZ, XS, EAB, and AP: methodology. HY, WZ, EB, and XS: conducting experiments. XS, WZ, and JB: statistical analysis of the results. XS, JB, EAB, MI, and HY: writing—original draft preparation. XS, JB, EAB, WZ, AP, MI, HY, and EB: writing—review and editing. All authors approved the submission.

## Conflict of Interest

The authors declare that the research was conducted in the absence of any commercial or financial relationships that could be construed as a potential conflict of interest.
